# Study of new practical ESR dosimeter based on carbonated hydroxyapatite and its dosimetric properties

**DOI:** 10.1371/journal.pone.0197953

**Published:** 2018-05-29

**Authors:** Ye Liu, Lei Ma, Junwang Guo, Guofu Dong, Jianbo Cong, Yunlong Ji, Jing Ning, Guoshan Yang, Ke Wu

**Affiliations:** Beijing Institute of Radiation Medicine, Beijing Key Laboratory of Radiation Biology, Beijing, P. R. China; North Shore Long Island Jewish Health System, UNITED STATES

## Abstract

The development of new dosimeters with good dosimetric properties is important for quality control in radiation applications. A new practical electron spin resonance (ESR) dosimeter based on carbonated hydroxyapatite that simulated the composition and structure of tooth enamel was specially synthesized. The synthesized material was investigated by transmission electron microscope, X-ray diffraction, fourier transform infrared spectroscopy and X-ray photo electron spectroscopy to confirm to the main composition of carbonated hydroxyapatite with CO_3_^2-^ successfully doped into the crystal lattice through optimizing the synthesis process of C/P molar ratio, pH value dynamical adjustment, annealing temperature and time. The dosimetric properties were systematically investigated by ESR spectroscopy. The results indicated that the radiation induced signal had a good dose response within a relatively wide dose range. The dose response was linear in the dose range of 0–400 Gy with a correlation coefficient of 0.9999 and had dose accumulative effect in the experimental dose range of 0–100 Gy. In a wider dose range up to 30 kGy, the dose response also presented linear feature in double-logarithmic coordinate system with a correlation coefficient of 0.9970. The dose detection limit was about 0.34Gy with a given probability of 95% confidence level depending upon a rigid calculation algorithm. The signal was extremely stable in the observation time of 360 days with a variation coefficient of 3.8%. The radiation sensitivity of the material showed no remarkable variation against photon energy from 662 KeV to 1.25 MeV and dose rate from 0.86 Gy/min to 12.17 Gy/min. The material showed more sensitive in lower photon energy range below 662 keV, which hint additional calibration may need when using in special photon energy condition. The preliminary results suggested that this newly developed dosimeter was potential to become a practical dosimeter that would expand the application fields of ESR dosimetry.

## Introduction

Ionizing radiation has been widely used in many fields. The development of new dosimeters with good dosimetric performance properties is important for quality control in radiation applications. Electron spin resonance (ESR) dosimetry is recognized as an attractive approach by detecting the radiation induced long-life free radicals in materials. As a typical example, alanine ESR dosimeter has become a standard method which has been applied in radiation process applications. However, to develop dosimeters that have better dosimetric properties, such as with lower dose detection limit and wider dose response range, more stable, good tissue equivalent etc., has always been a valuable attempt especially for the usage of quality control in clinical radiation applications.

Tooth enamel is an effective ESR dosimeter material that has been verified in the applications of dose assessment in various radiation events. Tooth enamel ESR dosimeter has some prominent advantages, such as wide dose response range, relatively simple measurement process, extremely stable radiation induced signal (RIS), characters of dose accumulation and being able to be recorded repeatedly.

The major composition of tooth enamel is carbonated hydroxyapatite (CHAP). The CHAP can produce stable paramagnetic center CO_2_^-^ free radical after irradiation which comes from the impurity substance of CO_3_^2-^ that substitutes OH^-^ or PO_4_^3-^ group or mixture in the apatite crystal [1–7]. The CO_2_^-^ free radical was then found to become as dose indicator. Inspired by the principle of ESR tooth dosimetry, the exploration of new dosimeter based on CHAP has been continuing all the time [8–14]. Synthetical method of CHAP was also got progress [15–18].

In recent years, L.M.de, Oliveira and his colleagues reported a series researches aimed to develop CHAP dosimeter suitable for clinical therapy use [2, 8–11]. The overall uncertainty of their experiment in the range of 2–100 Gy was around 1% at 95% confidence level. Besides features of stability and weak energy dependence, clear RIS signal of dose as low as even 0.5 Gy could be observed, which suggested lower dose detection limit might obtain. All these previous work suggests that it is worth and promising to continue exploring new and more practical ESR dosimetric materials of lower detection limit and wider dose range along this way for the usage of more fields.

Based on the work above especially the valuable attempt of L.M.de, Oliveira, this paper introduces a practical CHAP dosimeter that simulated the tooth enamel in composition (hydroxyapatite) and structure (the carbonate ion in the lattice). The material features, the dosimetric properties and optimization of the synthesis process were then explored systematically and discussed.

## Materials and methods

### Synthesis of the CHAP

**Fig 1:**
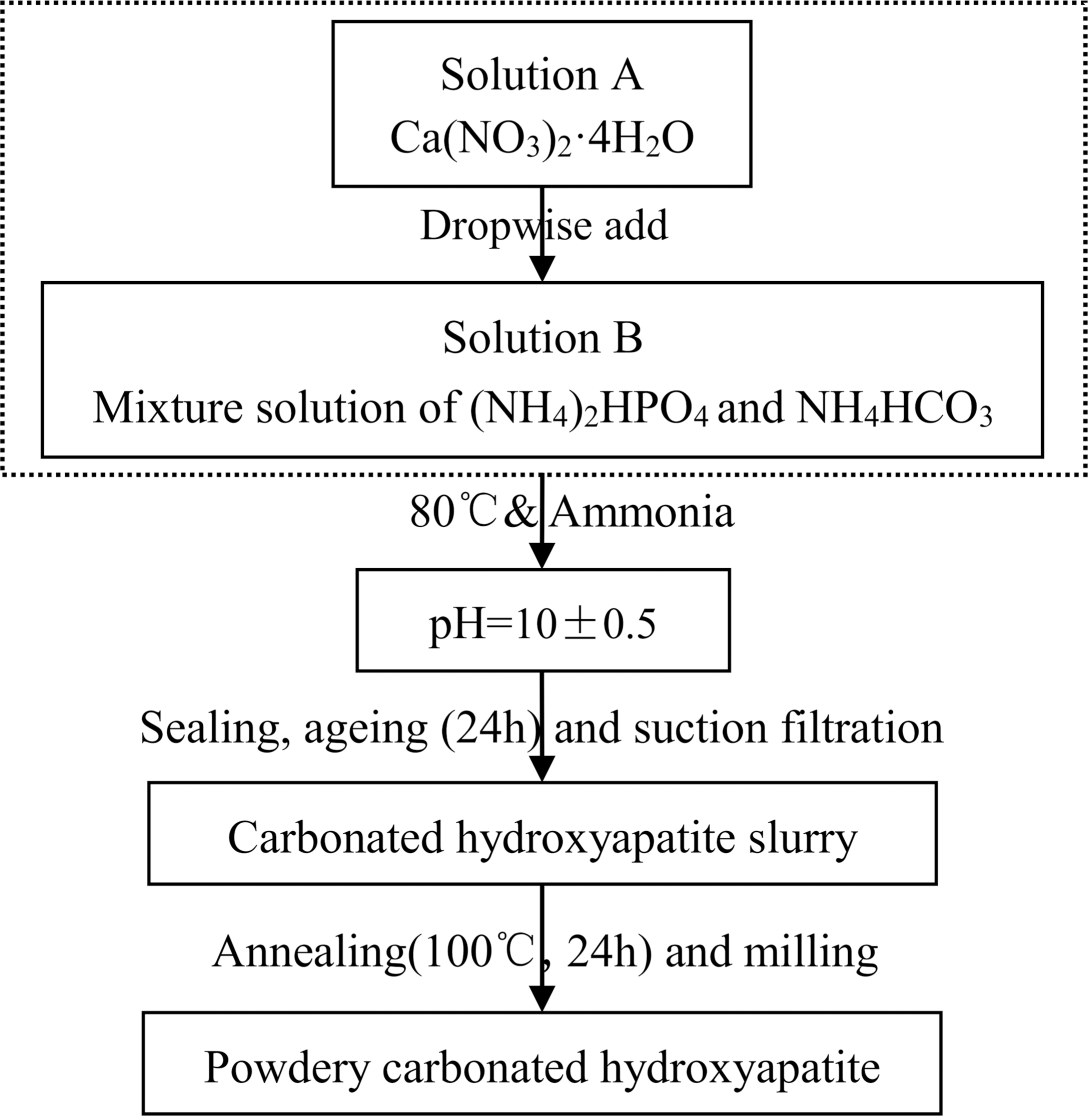
Synthesis flow chart of CHAP.

According to the principle of radiation induced free radical in CHAP [1–14], the doping effect of CO_3_^2-^ was most concerned when the synthesis program was trialed. The synthesis program established in this study was based on previously reported work involving a double-decomposition method described in literatures [19–21] and to which some improvements were made. The finally established program is indicated as the synthesis flow chart in Fig 1. The precipitation process of CHAP was kept stirring as the calcium nitrate tetrahydrate (solution A: 6.372 g of calcium nitrate tetrahydrate [Ca(NO_3_)_2_·4H_2_O] (Sinopharm Chemical Reagent co., LTD, Beijing, China) was dissolved in 600 ml of Deionized water to produce 0.027 mol of calcium ions Ca^2+^) was added dropwise (26.3ul/s with a modified medical infusion apparatus) by automatic titrator into the solution of ammonium hydrogen orthophosphate and ammonium bicarbonate (solution B: 2.377 g of ammonium hydrogen orthophosphate [(NH_4_)_2_HPO_4_] (Sinopharm Chemical Reagent co., LTD, Beijing, China) and 5.692g of ammonium bicarbonate [NH_4_HCO_3_] (Sinopharm Chemical Reagent co., LTD, Beijing, China) were dissolved in 1500 ml of deionized water to produce 0.018 mol of PO_4_^3-^ and 0.072 mol of CO_3_^2-^). After the drip was finished, the reaction was kept continuing stirring for 1h. Throughout the reaction, the pH was stabilized at 10±0.5 using ammonium hydroxide solution and the temperature controlled at 80°C.The resulting suspension was then aged for 24h at room temperature and washed with deionized water, annealed for 24 h at 100°C, milled last. Most of the following dosimetric experiments were carried out with powder CHAP samples. In order to inspect a lower dose detection limit, some of the dosimeter samples were shaped into cylinder pieces with a model (each tablet piece contains 60 mg CHAP powder) because the shaped dosimeter piece had better measurement precision than powder dosimeter to some extent (the standard deviation of the radiation induced signal from 10 Gy irradiated shaped dosimeters reduced by 32% than powder dosimeters as data shown in S1 Table).

### Carbonate content and dosimetric sensitivity

**Fig 2:**
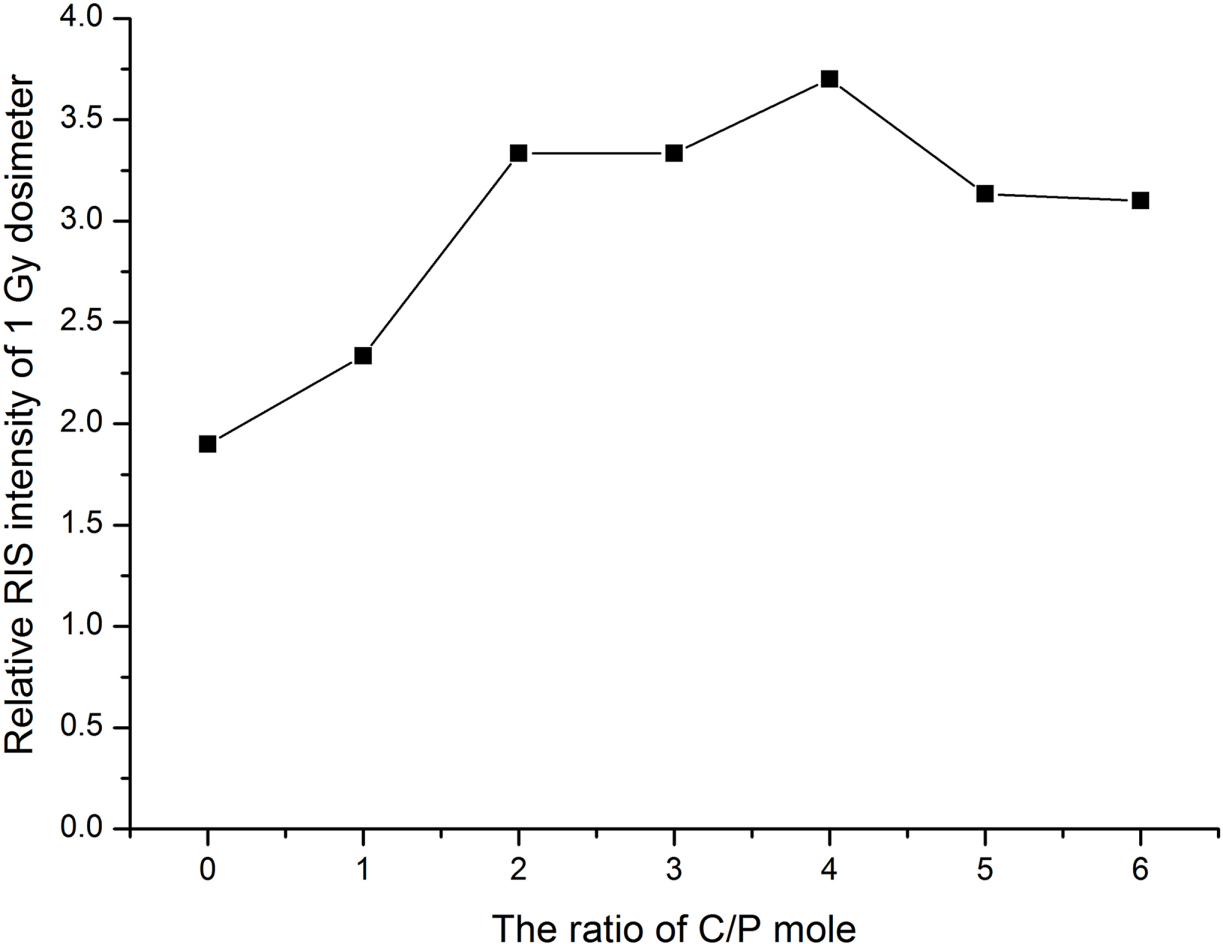
The change of RIS intensity of different molar ratios of C/P.

As the RIS comes from the free radial generated by the CO_3_^2-^ impurity in the CHAP crystal lattice after irradiation [1–14], the carbonate content should play an important role for the dosimetric sensitivity of this material used as dosimeter. The carbonate content was controlled by the molar ratio of C/P (NH_4_HCO_3_/(NH_4_)_2_HPO_4_) in the sample synthesis process. Therefore, different molar ratios of C/P at 0, 1, 2, 3, 4, 5 and 6 in solution B were trialed in this experiment. We evaluated the effect of the molar ratio of C/P by measuring the RIS intensities (1 Gy irradiated) against different molar ratios of C/P. The result is illustrated in Fig 2. Fig 2 which shows that more sensitive radiation property appeared around the molar ratio of C/P at 4. Thus we got a relatively satisfactory result in the followed dosimetric feature experiments.

### Sample characterization

The morphology and microstructure of CHAP were analyzed by Transmission Electron Microscope (TEM) on the instrument of Hitachi H-7650. The images were recorded at 200 kV in the bright field mode along with the corresponding diffraction patterns. The material composition and structure were studied by X-ray diffraction (XRD) on a Philips Analytical X-Ray B.V. with the use of Ni-filtered Cu Kα source In addition, typical apatites (PDF: 72–1243) was used as standard for comparison and inspection. The location of CO_3_^2-^ was analyzed by Fourier Transform infrared spectroscopy (FTIR), and the FTIR spectra in the range of 4000–400 cm^-1^ was recorded as KBr pellets with a VERTEX 80/80v FT/IR Spectrometer (Bruker).The CO_3_^2-^ doping state in the CHAP lattice was investigated by X-ray photo electron spectroscopy (XPS) of Thermo escalab 250XI electron spectrometer fitted with an Al Kα source (soft X-ray source at 1486.6 eV, which is non-monochromatic). The anode was operated at 150 W. The binding energy shifts due to surface charging which were corrected with using the C 1s level at 284.8 eV, as an internal standard.

### Sample irradiations

The total number of more than 270 dosimeters were irradiated in this experiment. Among them, 222 dosimeters were irradiated by ^60^Co γ-ray sources, 21 dosimeters were irradiated by 137Cs γ-ray sources, and 27 dosimeters were irradiated by X-ray machine. Most of the γ-irradiations were performed using the ^60^Co γ-ray sources at dose rate between 0.86 Gy/min to 12.17 Gy/min respectively according to the requirements of experiments. The phonton energy dependence experiments were carried out using ^60^Co and ^137^Cs γ-ray sources correspondence photon energy of 1.25 MeV (Average of 1.17 MeV and 1.33 MeV) and 662 keV, and X-ray machine YXLon MG325 (Narrow band spectrum serials, correspondence photon energy of 48 keV, 100 keV and 250 keV). The dose rate was measured with PTW UNIDOS dosimeter and Farmer-30012 0.6cc ion chamber.

### ESR measurements

ESR measurements were done with a Bruker A300 ESR Spectrometer operating at the X-band microwave frequencies. The ESR operational settings were: central magnetic field 350 mT, sweep width 20 mT, microwave power 10 mW, modulation frequency 100 kHz, scan time 60 seconds and time constant 40 milliseconds. The received gain parameter was adjusted to optimize the ESR signals.

### Data analysis

All the data analyses were performed using software Origin 8. Most of the statistic figures were created in orthogonal coordinate system except the dose response analysis in the wide dose range experiment of 1-30kGy as the orthogonal coordinate system was not fit to display explicitly all data in so wide data range. In this case, the double-logarithmic coordinate system was used therefore the ESR signal intensity (I) to the dose (D) response feature was fitted as Lg(I) = a×Lg(D)+b. The parameters a and b would be determined by experiment data.

## Results and discussion

### The material analysis

**Fig 3:**
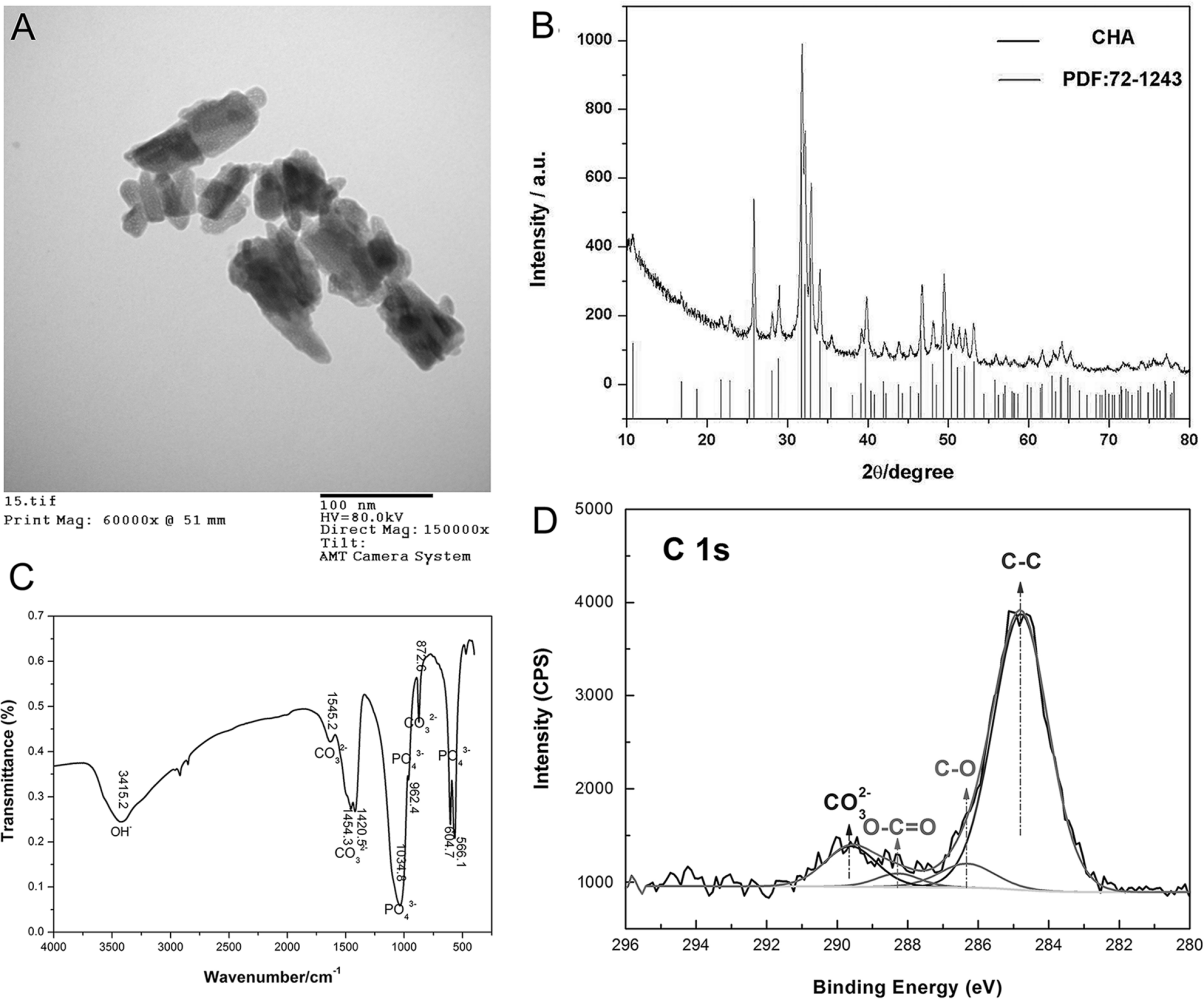
The results of material analysis. (A) TEM micrographs. (B) XRD analysis. (C) FTIR spectra. (D) XPS analysis.

The TEM micrographs of the CHAP dosimetric sample is shown in Fig 3A. The CHAP particle exhibited short and rod-like shape. The particle size and length were appropriate 25-45nm and 80-160nm respectively. The XRD spectrum (shown in Fig 3B) demonstrated that the dosimeter was mainly consisted of carbonated hydroxyapatite and this conclusion was further verified by the FTIR spectra (shown in Fig 3C) which indicated that CO_3_^2-^ had been invaded into CHAP intracell successfully because the vibrating peak of CO_3_^2-^ was singlet. The material exhibited antisymmetrical concertina motion of CO_3_^2-^ at 1545.2, 1454.3, 1420.5, and 872.6 cm^-1^, showing that the material was an AB-type carbonate-substituted hydroxyapatite [22]. The XPS spectra showed that CHAP sample were consisted of Ca, P, O, C and a small quantity of N, and CO_3_^2-^ was successfully doped into the material (Fig 3D).

### The dosimetric properties

#### Character of radiation induced ESR spectra

**Fig 4:**
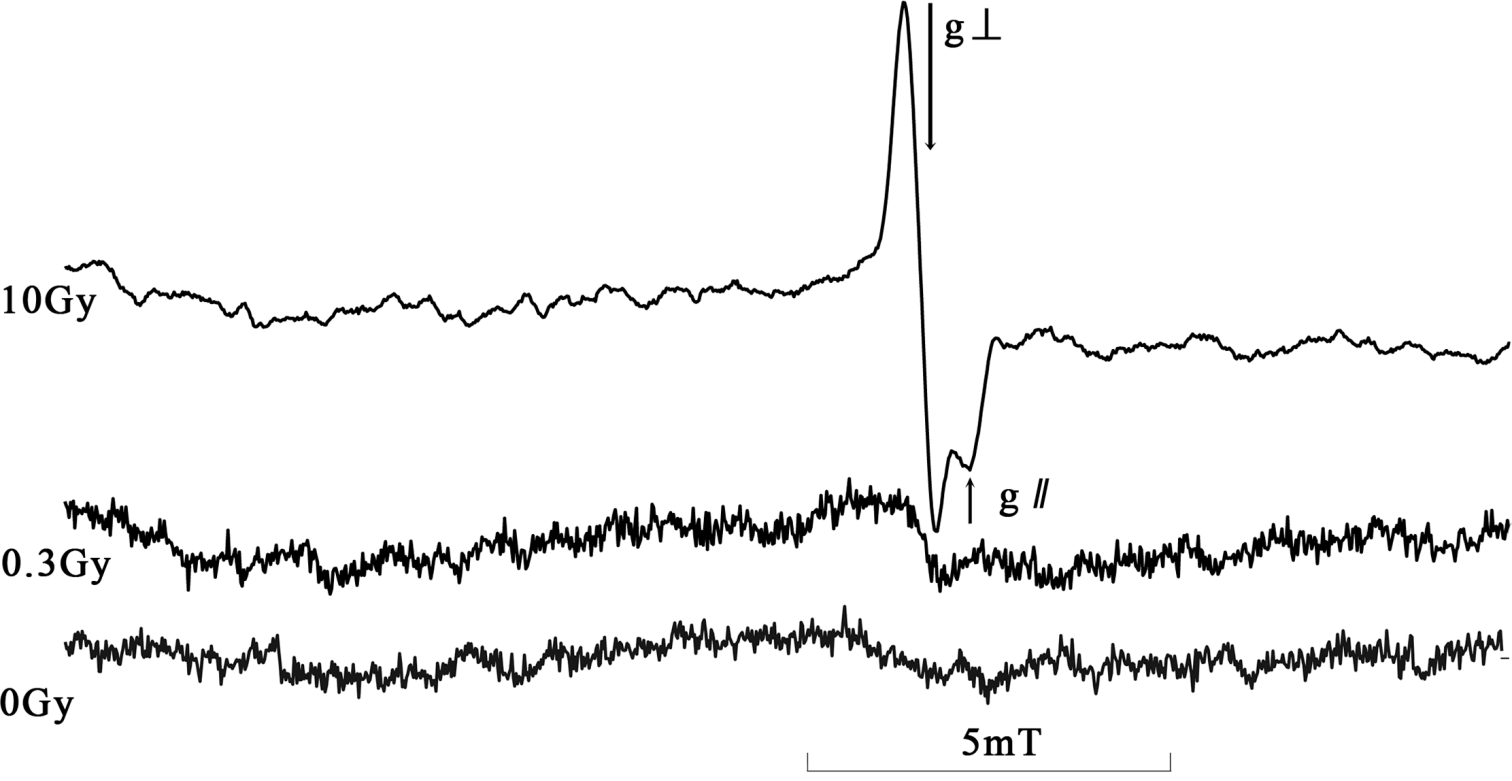
The ESR spectra of dosimeter irradiated at 0 Gy, 0.3 Gy and 10 Gy.

Fig 4 shows the typical ESR spectra of the CHAP samples unirradiated and irradiated at 0.3Gy and 10Gy. Radiation induced signal (RIS) could be observed in the spectrum at the g-values of g_⊥_ = 2.0022 and g_//_ = 1.9980, which correspond to CO_2_^-^ free radical [1, 11]. Slightly discernable spectrum component of RIS could be observed from the spectrum irradiated at a dose of 0.3Gy.

#### Background signals

Distribution of background signals was got by ESR measurements of twenty unirradiated samples from different batches. There were no remarkable signals that can be clearly discernible from the appearance of spectra of unirradiated samples as shown in Fig 4. We calculated the relative signal intensity in the same spectrum position as that of the RIS and got the background signal distribution was 2.03±0.16 with coefficient of variation of 7.9%.

#### Dose response feature

**Fig 5:**
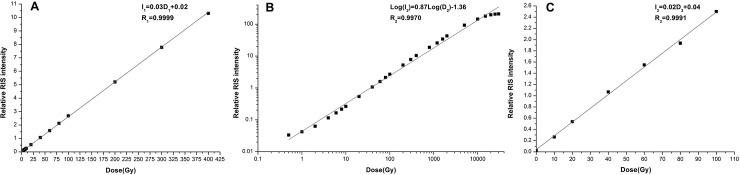
The dose response curve. (A) The RIS intensity against radiation dose (0–400 Gy). (B) The RIS intensity against radiation dose (1Gy-30 kGy). (C) The RIS intensity against accumulated radiation dose (0–100 Gy).

Fig 5A shows the dose response of RIS intensity to radiation dose. The RIS intensity increased linearly in the dose range of 0–400 Gy. The fitting curve function was I_1_ = 0.03D_1_+0.02 (taking I as the RIS intensity and D as the radiation dose) and the linear coefficient R_1_ = 0.9999 when the dose in the range of 0–400 Gy.

To evaluate the adapt ability of more applications, dose response in a wider dose range was further investigated in the range of 1Gy-30 kGy. As the dose range covers 4 orders of magnitude, the orthogonal coordinate system will not fit to display explicitly all data in so wide data range. So the ESR signal intensity and dose were illustrated in double-logarithmic coordinate system as shown in Fig 5B. The fitting curve function was Lg(I_2_) = 0.87×Lg(D_2_)-1.36.

#### Dose accumulation feature

Fig 5C shows the dose accumulation feature of RIS intensity from samples irradiated additively in the dose range usually being used in clinical application. When the dose increased from 0 Gy to 100 Gy, the RIS intensity also increased linearly. The fitting curve function was I_3_ = 0.02D_3_+0.04, and the linear correlation coefficient R_3_ = 0.9991.

#### The dose detection limit

Although typical spectrum of discernible RIS could be observed from samples irradiated at the dose of 0.3Gy, to determine objectively the dose detection limit of the dosimeter, algorithm for the calculation of detection limit was referred as the method described by P Fattibene et al [23]. The detection limit (DL) specifies the minimum (true) value of the measurement which can be detected with a given probability of error using the measuring process in question (ISO, 1998). The detection limit is subsequently related to the inherent detection capability of a method (IUPAC, 1995). We made 20 measurements and calculated the detection limit. The signal distribution was 2.03±0.16 (background) and 23.43±0.19 (irradiated samples) respectively. The final calculated dose detection limit result was 0.34Gy with a given probability of 95% confidence level.

#### Dose rate and photon energy dependence

**Fig 6:**
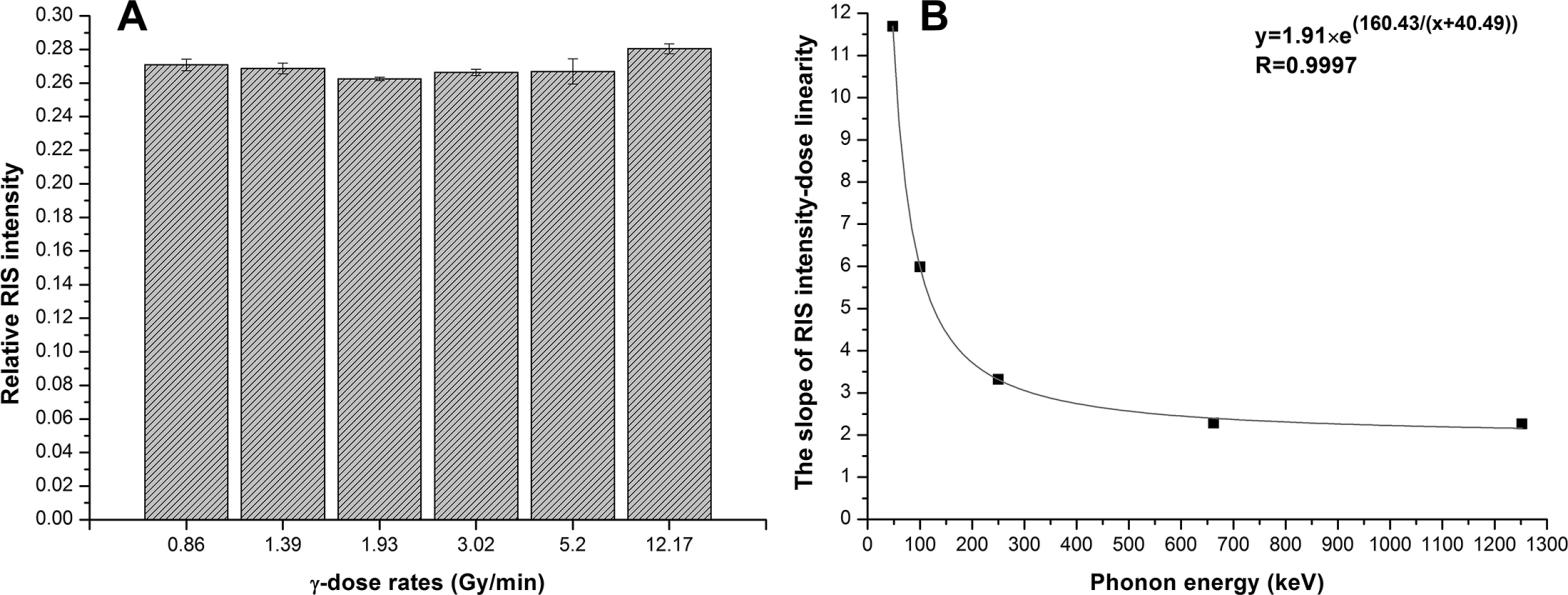
The dose rate effect (A) and photon energy dependence (B) of dosimeters.

To evaluate the dose rate effect on the ESR signal intensity of the irradiated CHAP dosimeters, six groups of dosimeters were irradiated at the same dose of 10 Gy but in different dose rate of 0.86 Gy/min, 1.39 Gy/min, 1.93 Gy/min, 3.02 Gy/min, 5.20 Gy/min and 12.17 Gy/min by ^60^Co gamma source respectively. Fig 6A shows the RIS intensity of CHAP dosimeters at six different dose rates. The coefficient of variation was 2.3% for the six groups. We evaluated the intrinsic measurement error of the method by a standard sample of stable free radical (strong pitch, Bruker) and had a coefficient variation of 1.9%. Therefore, there was no significant dose rate dependency could be observed within the dose rate range of this experiment. Some acceptable alternating as shown in Fig 6A might come from ESR measurement error or the uncertainty of irradiation process with different dose rates.

To evaluate the photon energy effect on the ESR-signal intensity of the dosimeter, another five groups of dosimeters were irradiated by different phonon energy between 48 keV~1.25MeV. Among them, 48 keV, 100 keV and 250 keV groups were irradiated by X-ray machine, 662 keV group was irradiated by ^137^Cs source, and 1.25 MeV group was irradiated by ^60^Co source respectively. We took the dose response sensitivity as an evaluation index of the photon energy dependence character. The result is shown in Fig 6B. When the energy was between 662 keV-1.25 MeV there was no significant change. While when the phonon energy was lower than 662 keV the dose response sensitivity increased obviously. The results indicated that the effect of photon energy could be ignored for energy above 662 keV. However, specific calibration is needed according to different photon energy when applying in the lower energy range especially for most x-ray applications. This result also partially coincided with that reported by L.M.de Oliveira [2].

#### Signal stability

**Fig 7:**
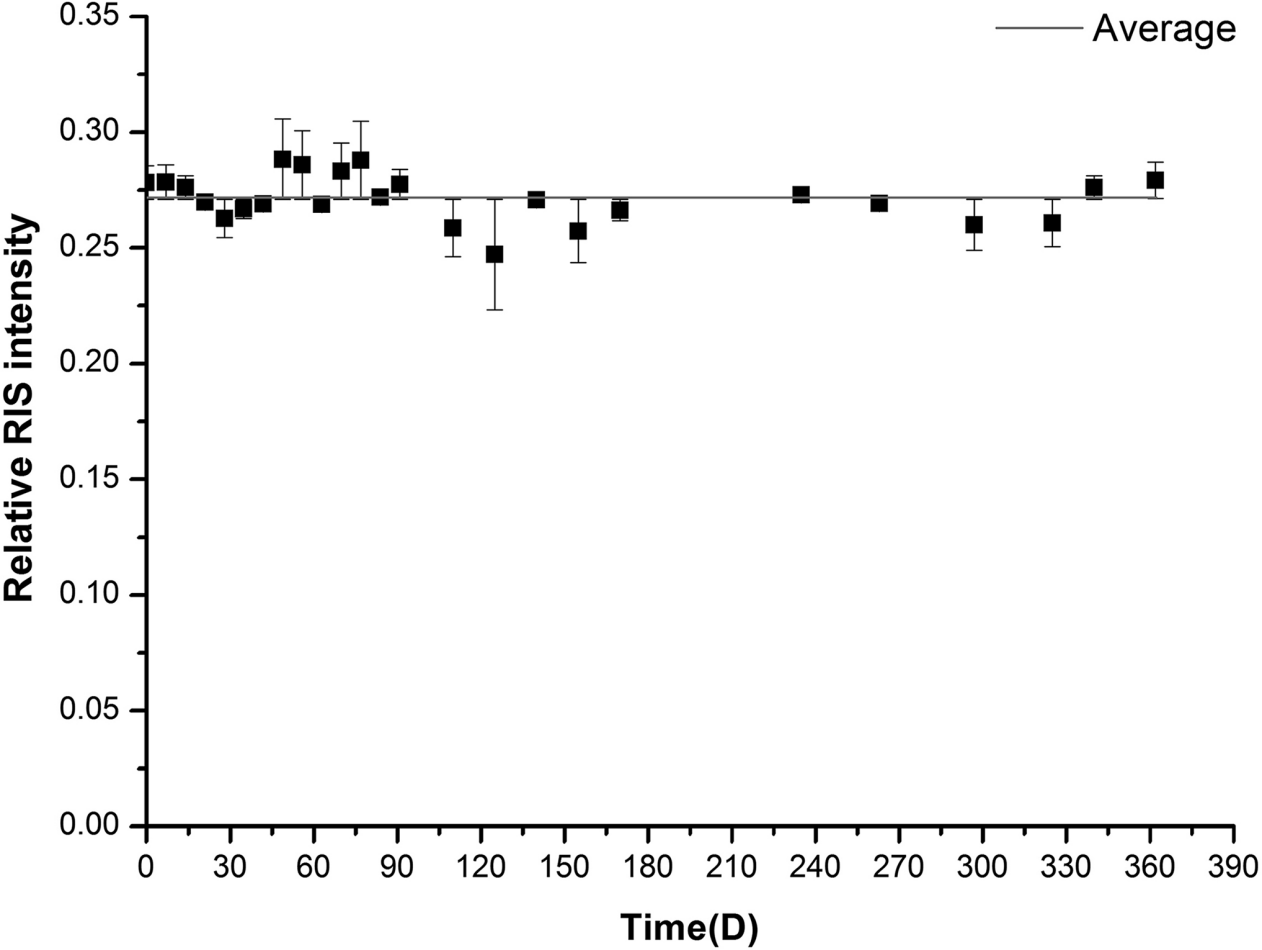
The RIS intensity against time (Days) after irradiation.

Dosimeters irradiated at a dose of 10 Gy using ^60^Co gamma source were stored in ordinary laboratory condition and measured in different time repeatedly. Fig 7 shows the changing of RIS signal intensity in different time after radiation. There was no significant decaying tendency within the whole observing period of 360 days. The average coefficient of variation was 3.8%.

In order to investigate some other conditions that may be met in routine usage and may influence the stability, we also studied the signal variation at regular intervals of time when the dosimeters were stored in different temperatures, different relative humidity and constant illumination conditions. The results showed: The coefficient of variation were 2.5%, 2.3% and 2.9% in relative humidity of 12%, 36% and 76%, respectively. When the storage temperature were 4°C, 20°C and 40°C, the coefficient of variation were 2.8%, 3.8% and 3.9%, respectively. The coefficient of variation was 3.8% when storing the samples in a constant illumination condition. All the variations in this experiment conditions were in the acceptable level comparing to the already used dosimeters (ISO/ASTM 51607:2013).

## Conclusions

Some specific dosimetric performances can be concluded for the developed CHAP dosimeter in this work. (1) the radiation induced signal (RIS) had a relatively wide range of dose response; (2) The RIS also showed relatively lower dose detection limit (about 0.34 Gy) among the same kind of dosimeter at present, such as alanine dosimeter. (3) It also has good linear accumulative dose response in the experiment range of 0-100Gy. (4) The signal stability of this dosimeter was suitable for most application fields. Considering the experimental uncertainties, there was no significant signal attenuation during the whole experiment time even under different environmental conditions that may be met in routine use. Therefore, it showed potential applicability of dose monitoring for both cancer clinical radiotherapy (usually several Gy for single irradiation and tens of Gy totally) and radiation processing field (~ kGy). While, it is still need to be improved for some types of measurements such as pacemaker.

Comparing to the dosimetry features with previously developed ESR dosimeter, the major features of this dosimeter exhibit lower detection limit, better signal stability. In addition, different synthetic method was used.

It was just a preliminary trying to optimize the dose sensitivity by controlling the C/P molar ratio therefore adjusting the CO_3_^2-^ doping in CHAP, which suggested something new improvement of the previous method. Besides that, some other improvements of the synthesis process were also used, such as adjusting the pH value in a dynamical dropwise way therefore to maintain the pH value in a relatively constant level during the whole synthesis process that was controlled in an extremely slow dropwise speed of 26.3ul/s. In addition, a relatively lower annealing temperature but longer annealing time was carried on after the synthesis process. Because additional signal was found in the spectra of sample with high annealing temperature. These improvements obviously benefited for controlling the product quality and for insuring the correct amount of CO_3_^2-^ to be doped into the material crystal lattice. And according to the results, some beneficial suggestions could also be obtained that may further improve the dosimetric properties of the dosimeter such as finding more effective doping methods or doping position of CO_3_^2-^ in the material.

There remains a problem of the present work that the standard deviations among different sample batches exist some inconsistency (as shown in S2 Table). The possible reasons for that may be as: Firstly, the long span of production and measurement of the 6 different batches (the whole process spanned 3 months). During this period, the measuring condition and operation process could not be completely kept consistency. Secondly, the amount of adhesion on tube wall might also be different when samples were repeatedly filled into sample tube. However, The standard deviation among the mean values of the 6 batches is only about 2.1%, which means the accuracy of measurement is not influenced much more than that caused by other factors such as temperature or humidity (almost above 3%). Therefore we think it is at an acceptable level. Anyhow, the difference of measuring precision among batches really remains a problem that needs special attention in the future research and application.

The preliminary results of present work suggested that the CHAP developed in this work has merited characteristics potential to be developed as a practical dosimeter to expand the application fields of ESR dosimetry.

## Supporting information

S1 Table The RIS intensities of dosimeters irradiated by 10 Gy. (DOCX)

S2 Table The RIS intensities of dosimeter materials from 6 batches irradiated by 10 Gy. (DOCX)
