# Supplementary material for: Study of new practical ESR dosimeter based on carbonated hydroxyapatite and its dosimetric properties
Source: PLoS One. 2018 May 29;13(5):e0197953. doi: 10.1371/journal.pone.0197953 (PMC5973591; doi:10.1371/journal.pone.0197953)
Supplement: S1 Table — (DOCX) [file pone.0197953.s001.docx]

S1 Table. The RIS intensities of dosimeters irradiated by 10 Gy.

| The times of measurements | The RIS intensities of shaped dosimeters (×10^5^) | The RIS intensities of powder dosimeters (×10^5^) |
| --- | --- | --- |
| 1 | 25.67 | 25.90 |
| 2 | 25.80 | 25.76 |
| 3 | 25.83 | 25.61 |
| 4 | 25.50 | 25.49 |
| 5 | 25.80 | 25.24 |
| 6 | 25.84 | 25.31 |
| 7 | 25.56 | 25.24 |
| 8 | 25.32 | 25.26 |
| 9 | 25.67 | 25.19 |
| 10 | 25.78 | 25.25 |
| $\bar{x}\pm s$ | 25.68 ± 0.17 | 25.43 ± 0.25 |
