# Supplementary material for: Study of new practical ESR dosimeter based on carbonated hydroxyapatite and its dosimetric properties
Source: PLoS One. 2018 May 29;13(5):e0197953. doi: 10.1371/journal.pone.0197953 (PMC5973591; doi:10.1371/journal.pone.0197953)
Supplement: S2 Table — (DOCX) [file pone.0197953.s002.docx]

S2 Table. The RIS intensities of dosimeter materials from 6 batches irradiated by 10 Gy.

| Batches | RIS intensities ($\bar{x}\pm s$, ×10^5^) |
| --- | --- |
| 1 | 24.55±0.51 |
| 2 | 26.43±1.02 |
| 3 | 24.98±0.46 |
| 4 | 26.11±1.36 |
| 5 | 25.47±0.25 |
| 6 | 27.38±0.08 |
